# A Comprehensive Review of the Role of UV Radiation in Photoaging Processes Between Different Types of Skin

**DOI:** 10.7759/cureus.81109

**Published:** 2025-03-24

**Authors:** Gurjasan Brar, Anoop Dhaliwal, Anupjot S Brar, Manasa Sreedevi, Yasmin Ahmadi, Muhammad Irfan, Rebecca Golbari, Daniela Zumárraga, Dana Yateem, Yuliya Lysak, Yozahandy A Abarca-Pineda

**Affiliations:** 1 Internal Medicine, St. George's University School of Medicine, True Blue, GRD; 2 Internal Medicine, St. George's University School of Medicine, Toronto, CAN; 3 Internal Medicine, University Hospitals Leicester, Leicester, GBR; 4 Internal Medicine, Royal Shrewsbury Hospital, Shrewsbury, GBR; 5 Internal Medicine, Technion American Medical School, Haifa, ISR; 6 Internal Medicine, Universidad San Francisco de Quito, Quito, ECU; 7 Medicine, St. George's University School of Medicine, True Blue, GRD; 8 Internal Medicine, Hospital Médica Sur, Mexico City, MEX

**Keywords:** fitzpatrick skin types, melanin, photoaging, photoprotection, sunscreen and uv protection, ultraviolet radiation (uvr)

## Abstract

Ultraviolet (UV) radiation significantly contributes to photoaging, with its effects varying among different Fitzpatrick skin types. Light skin (Types I-III) has a natural sun protection factor (SPF) of only 3.3, making it particularly vulnerable to DNA damage, collagen degradation, and skin cancer. Darker skin (Types IV-VI) has a natural SPF of 13.4, providing greater photoprotection while elevating the risk of post-inflammatory hyperpigmentation and delaying skin cancer diagnosis.

UVA penetrates deep into the dermis, promoting collagen degradation, whereas UVB causes DNA mutations, increasing the risk of cancer. Eumelanin in darker skin mitigates oxidative stress, while pheomelanin in lighter skin functions as a pro-oxidant, increasing vulnerability to photoaging. Although incidence rates are lower, melanoma is identified at more advanced stages in those with darker skin, resulting in poorer outcomes.

Protective measures, such as broad-spectrum sunscreens, antioxidants, and hydration, are crucial for all skin types but necessitate customized strategies. Individuals with lighter skin benefit from SPF 50+ and DNA-repairing compounds, whereas those with darker complexion necessitate SPF 30-50 and pigmentation-focused skincare. Comprehending the biological mechanisms and variations in UV damage facilitates the creation of customized photoprotection solutions, enhancing skin health and mitigating long-term UV-related issues for all skin types.

## Introduction and background

Human skin shows different variations, greatly influenced by genetic, environmental, and lifestyle factors. The Fitzpatrick skin type classification developed by Dr. Thomas Fitzpatrick in 1975 categorizes skin types into six distinct categories, ranging from Type I (very light skin that always burns and never tans) to Type VI (deeply pigmented skin that rarely burns and tans easily) [1,2]. 

This helps in assessing individual sensitivity to ultraviolet (UV) radiation and the associated risk of photoaging, a process defined by premature aging of the skin due to chronic UV exposure which can manifest as wrinkles, loss of elasticity, uneven pigmentation, and other structural changes in the skin. The various molecular mechanisms underlying photoaging include degradation of extracellular matrix proteins like collagen, increased oxidative stress, and chronic inflammation [3,4]. Due to lower melanin levels, light-skinned individuals (Fitzpatrick Types I-III) are particularly vulnerable to UV-induced damage, which provide limited photoprotection. Conversely, individuals with darker skin types (Fitzpatrick Types IV-VI) benefit from higher melanin concentrations that offer greater natural protection but face unique challenges such as post-inflammatory hyperpigmentation and uneven skin tone [5,6]. These differences mark the importance of studying UV sensitivity and photoaging processes across diverse skin types.

These diverse effects of photoaging in skin colors have garnered increasing attention, emphasizing the need for tailored approaches to prevention and treatment. Dark-skinned individuals may exhibit fewer wrinkles and sagging due to increased dermal density, but they are prone to pigmentation disorders and textural changes [7]. Additionally, UV exposure has unique implications for darker skin, where conditions like photoaging and photo pigmentation manifest differently compared to lighter skin. Darker-skinned individuals have lower photo carcinogenesis risk but face challenges like polymorphous light eruption and melasma, which necessitate specialized preventive measures. Broad-spectrum sunscreens addressing UVA, visible light, and infrared radiation are critical, along with antioxidants to mitigate free radical damage [8].

UVA and UVB, the two primary components of UV radiation, contribute distinctly to photoaging. UVA penetrates deeply into the dermis, causing oxidative stress and collagen breakdown, while UVB primarily affects the epidermis, inducing DNA damage and inflammation [3,4]. Both mechanisms activate matrix metalloproteinases (MMPs), enzymes responsible for collagen degradation, and suppress DNA repair pathways, exacerbating aging processes [9]. Chronic UV exposure further accelerates skin aging by promoting inflammation and immunosuppression, mechanisms noted in light-skinned populations but less thoroughly explored in darker-skinned individuals [10].

A comprehensive understanding of UV-induced photoaging across different skin types is essential for personalized dermatological care. Insights into these processes enable the development of tailored prevention strategies, such as sunscreens optimized for specific Fitzpatrick types, and targeted therapeutic interventions [11]. This narrative review aims to synthesize existing knowledge on the role of UV radiation in photoaging, with a focus on the differential impacts on light and dark skin types. By exploring key molecular mechanisms and clinical implications, this review seeks to contribute to the broader understanding of skin aging and inform strategies for improved skin health management.

## Review

Skin pigmentation and UV radiation 

Melanin pigments are synthesized in the epidermis by melanocytes, specialized cells originating from the neural crest. During embryogenesis, these cells migrate as melanoblasts into the epidermis and hair follicles through the mesenchyme. The process of melanogenesis is highly complex, involving numerous regulatory agents and pathways that operate within melanocytes and in coordination with other skin cell types [5]. Positioned at the dermal-epidermal junction, each interfollicular melanocyte extends its dendrites to connect with around 40 keratinocytes, forming what is known as the epidermal melanin unit [12]. 

In melanocytes, melanin is produced within unique, specialized, membrane-bound structures known as melanosomes. Differences in inherent skin pigmentation are primarily due to variations in the type and amount of melanin, as well as in the size, number, distribution, and behavior of melanosomes within the epidermal melanin unit. These differences are not linked to variations in melanocyte density, as the number of melanocytes is relatively consistent across ethnic groups [13-15]. 

Melanin acts as a protective shield that disperses ultraviolet radiation (UVR) and limits its infiltration through the epidermis. It is estimated that the epidermis of dark skin has a natural sun protection factor (SPF) of 13.4, compared to light skin, which has an SPF of 3.3 [16]. This is generally believed to stem from variations in the size of melanocytes and the distribution and packaging of melanosomes [17]. Melanocytes generate two primary forms of melanin from dihydroxyphenylalanine precursors: the yellow-red pheomelanin and the black-brown eumelanin. Eumelanin offers better photoprotective qualities, and the ratio of eumelanin to pheomelanin affects skin type [18]. Variations in the size, quantity, and arrangement of melanosomes, along with the type of melanin they produce and their melanin content - which varies from 17.9% to 72.3% - are what define skin pigmentation [19]. Skin of color features notably larger melanocytes, a greater eumelanin-to-pheomelanin ratio, and melanosomes that are individually dispersed within keratinocytes instead of clustered together. This arrangement enhances the efficiency of absorption [20]. The enhanced melanin levels and distribution in the epidermis naturally protect against sunlight by absorbing and scattering two to five times more UV photons. Consequently, with the same exposure to ultraviolet radiation, individuals with lower epidermal melanin levels will experience more sunburn and less tanning compared to those with higher melanin levels [3]. Additionally, there are variations in melanosome breakdown based on skin type. In dark skin, melanosomes are less susceptible to breakdown by lysosomal enzymes and maintain their integrity across all epidermis layers [19,21]. In fair skin, melanosomes are broken down and only continue to exist as "melanin dust" within the upper layers beyond the base [18]. 

The skin exhibits significantly greater protection against DNA damage induced by UV radiation [22]. Fajuyigbe et al. demonstrated that both light and dark cyclobutane pyrimidine dimers (CPDs) were absent in regions with high melanin concentrations, such as the basal layer of Type VI skin [23]. The study's authors also noted that cellular repair processes for CPDs following UV exposure were comparable between lighter and darker skin types [23]. However, the higher melanin concentration in darker skin appears to provide an initial defense against CPD formation. Additionally, DNA strand breaks caused by UVA were observed more frequently in individuals with lighter skin [19,24]. Skin pigmentation plays a crucial role in photoprotection, as melanin not only absorbs UV radiation but also has antioxidant properties and the ability to neutralize free radicals [25]. This protective function is mainly attributed to eumelanin, whereas pheomelanin, which is more prevalent in lighter skin, acts as a pro-oxidant and lacks the ability to prevent CPD formation [22]. Consequently, eumelanin in darker skin types plays a key role in reducing UV-induced DNA damage in keratinocytes [24,26]. Furthermore, studies have shown that DNA damage repair mechanisms are more effective in skin of color than in lighter skin types [24]. Lopez et al. identified a fundamental difference in how melanocytes from melanin-rich and lighter skin respond to UVB exposure [25]. In melanin-rich skin, melanocytes exhibited enhanced melanogenesis due to increased cellular differentiation, while melanocytes in lighter skin showed proliferative activity without a corresponding increase in melanogenesis, indicating a lower level of protection against potential UVB-induced damage [27].

The ability of epidermal melanin to absorb UV radiation is considered one of the most vital natural defenses against the harmful effects of UVA and UVB. This largely explains why individuals with darker skin tend to show signs of photoaging much later in life compared to those with lighter skin tones [28]. Following UV exposure, melanin production typically increases as a protective response to minimize the risk of photoaging and skin cancer. This tanning mechanism has been linked to the induction of oligonucleotides containing thymine dinucleotides (pTpT), which are generated as a result of UV-induced damage (Table 1) [29].

**Table 1:** Comparison of Skin Pigmentation and UV Radiation Effects Table Credits: Daniela Zumárraga UV: ultraviolet, SPF: sun protection factor

Feature	Light Skin	Dark Skin
Melanin Type	More pheomelanin	More eumelanin
Melanin Distribution	Clumped melanosomes	Individually dispersed melanosomes
SPF Equivalent	~3.3	~13.4
UV Absorption	Less UV absorption	More UV absorption
DNA Protection	Lower protection, higher DNA damage	Higher protection, less DNA damage
Photodamage Response	More sunburn, less tanning	Less sunburn, more tanning
Melanosome Breakdown	Faster degradation	Slow degradation, persists across all epidermal layers

Sensitivity to UV radiation 

UV radiation from sunlight is a potent environmental risk factor with a considerable impairing influence on human health, including skin health [30]. Figuring out just how much of a UV dosage poses a risk is also dependent on skin pigmentation-which is an important determinant of the extent to which UV rays permeate the skin and inflict biological harm. People with lighter skin are more vulnerable to the negative consequences of UV radiation, whereas those with darker skin receive some inherent safeguard as a result of the increased levels of melanin in their skin [31,32]. However, both skin types experience distinct challenges and risks regarding UV exposure. 

Light-Skinned Individuals

Individuals with Fitzpatrick skin types I-III possess a higher susceptibility to UV radiation [1]. This is mainly because their skin contains less melanin, which has limited protection against the effects of UV exposure. Melanin absorbs UV and decreases its penetration deep through skin layers, protecting them from sunburn, DNA damage, and skin cancer. Individuals with lighter skin types have less melanin and therefore less protection from this type of photodamage from UV light [33].

Light-skinned individuals are at a higher risk of melanoma and skin cancers [33]. Basal cell damage occurs in lighter skin along with vitamin D synthesis, which explains the greater incidence of skin cancer [34]. Skin cancers can be fatal and are highly prevalent [35]. The main cause is the accumulation of mutations in genomic DNA after exposure to ultraviolet radiation.

Skin cancers are most common in lightly pigmented individuals who suffer chronic or high episodic doses of strong UVR in geographical areas distant from their ancestral homelands [36]. It has been hypothesized that pigmentary changes may have occurred following the migration of lightly and moderately pigmented populations, such as the so-called “Ancestral North Indians” into the high-UVR regions of the Indian subcontinent, and similarly, East Asians into the high-UVR environments of Central and mountainous South America [36]. However, these claims remain speculative and would benefit from further support through genetic and anthropological studies.

Higher Susceptibility to Sunburn and DNA Damage

Sunburn is the most common and almost immediate result of excessive exposure to UV radiation, especially in individuals with light-colored skin. Sunburn is caused by damage to the DNA in skin cells from UV radiation, resulting in inflammation, erythema (redness), and pain [37]. The immune response of the body to this damage leads to peeling and desquamation of the skin as it tries to get rid of the damaged cells. Less melanin present in the skin leads to a higher incidence of sunburn among individuals with fair skin, particularly those who are unprotected against UV radiation, when compared with people of darker skin types [37].

Lighter skin individuals have more pheomelanosomes which are less protective than eumelanin, which is found in darker skin and is more protective of UV radiation. These melanosomes found in lighter skin are found in the lower layers of the epidermis and are more quickly degraded. Pheomelanin, when exposed to ultraviolet radiation with a wavelength of 320-400 nanometers provides lower photoprotection [38].

The damage occurs not just on a cosmetic level; UV radiation can cause direct harm to your skin through the development of thymine dimers at the DNA level, which is the primary driving force behind the development of skin cancer when not repaired by the body's own DNA repair mechanisms. These mutations can lead to the formation of other types of skin cancer, such as basal cell carcinoma, squamous cell carcinoma (SCC), and the most malignant variety, melanoma [6]. Individuals with light skin are especially susceptible to these cancers, most notably melanoma, which is closely associated with having had severe sunburns during childhood and adolescence [39]. Additional studies have reported that those with fair skin are at higher risk for melanoma based on the lifetime accumulation of UV-induced DNA damage [30].

Increased Risk of Skin Cancers

Individuals with lighter skin tones face a heightened risk of developing non-melanoma skin cancers, including basal cell carcinoma and squamous cell carcinoma, as well as melanoma, due to prolonged exposure to UV radiation [40]. Basal cell carcinoma, the most common form of skin cancer, is primarily linked to extended sun exposure, which leads to DNA mutations in the basal cells of the epidermis. Similarly, squamous cell carcinoma can arise from chronic UV-induced damage to squamous cells, with both types being more frequently observed in individuals with fair skin [40]. Although less common than basal and squamous cell carcinomas, melanoma is the most deadly form of skin cancer and is strongly correlated with intermittent UV exposure, often resulting from sunburns [34].

Recent studies have highlighted the significance of early detection in fair-skinned individuals, where skin cancer tends to be diagnosed earlier, thanks to a more visible appearance on lighter skin. For instance, a higher incidence of melanoma in these populations can prompt early recognition of irregular moles or growths, leading to early-stage diagnosis and improved survival rates [41]. Nonetheless, melanoma incidence continues to rise in lighter-skinned populations worldwide, particularly in regions with high sun exposure, such as Australia and southern Europe [36].

In contrast, individuals with darker skin tones have significantly lower rates of skin cancer, due in part to the photoprotective effects of increased melanin. However, when skin cancers do occur in darker-skinned individuals, they are often diagnosed at more advanced stages, leading to poorer outcomes. This disparity may stem from reduced awareness, atypical presentation, and delayed diagnosis. Acral lentiginous melanoma, a subtype not strongly associated with UV exposure, is more common in people with darker skin and typically appears on the palms, soles, or under the nails, areas less frequently examined during routine skin checks [42]. Therefore, raising awareness and improving early detection strategies across all skin types remains critical to reducing the burden of skin cancer globally.

Need for Sunscreen and UV Protection in Light-Skinned Individuals

Higher dependence on sunscreen, protective clothing, and regular skin health checks are recommended to mitigate UV damage and cancer risk, as light-skinned individuals are more susceptible to UV radiation damage due to reduced melanin skin content [43]. Chronic exposure to UV radiation causes photoaging of the skin. Primarily, there are two types of UV rays: UVA and UVB. UVA rays can penetrate more deeply into the skin, causing the breakdown of collagen and elastin fibers, leading to wrinkles, fine lines, and skin laxity [43]. Whereas UVB is less penetrative but contributes to sunburn, it has a significant role in skin cancers (basal cell carcinoma, squamous cell carcinoma, and melanoma) [44,45]. Studies have shown that UV radiation accelerates the breakdown of extracellular matrix components, such as collagen and elastin, which contribute to the aged look of skin (saggy, rough texture, and loss of firmness) [44].

Chronic exposure to UV radiation damages DNA, leading to mutations in skin cells and increasing the risk of malignancy [43]. The reduced melanin content in the skin limits its natural defense against UV radiation by absorbing and dissipating the disruptive energies from UV rays and leading to elevated levels of oxidative stress in the skin [45]. Thus, highlighting the necessity of sunscreen and other forms of UV protection. 

Sunscreen is critical in preventing photoaging and mitigating the risk of UV-induced skin damage within the heightened sensitivity of light-skinned individuals. Sunscreen is an added barrier that either absorbs or reflects UV radiation, protecting the skin from harmful exposure [46]. Broad-spectrum sunscreens that protect against UVA and UVB rays are recommended for optimal protection [47]. Sunscreen is rated based on the SPF, which indicates the level of protection from UVB radiation [47]. An SPF of 30 or higher is generally considered adequate for daily use, though higher SPFs are often recommended for prolonged sun exposure or during activities like swimming or exercising outdoors [46]. Routine sunscreen application prevents photoaging and can significantly reduce the incidence of sunburns [46]. Also, sunscreen may preserve the skin's elasticity and mitigate the development of skin cancer, minimizing the effects of visible photoaging [46]. 

Furthermore, light-skinned individuals should wear protective clothing (long-sleeved shirts, hats, and sunglasses) combined with sunscreen to minimize direct sun exposure [31]. Clothing made of innovative fabrics with UV-blocking capabilities is an effective physical barrier in preventing UV radiation, skin damage, and photoaging [31].

Dark-Skinned Individuals

In contrast, Fitzpatrick skin types IV-VI are darker skin types, which have relatively higher amounts of melanin. Melanin absorbs and scatters UV radiation, offering natural protection against the sun's damage. Consequently, dark-skinned people have less likelihood of getting sunburn, DNA injury and skin cancer compared to light-skinned individuals [32].

Darker skin is more resistant to UV damage (photocarcinogenesis and photoaging) compared to lighter skin [32]. Darker skin has more eumelanin which is more protective than pheomelanin, which is seen in lighter or red-haired skin [32]. Due to this, dark-skinned individuals are less likely to get sunburned compared to light-skinned individuals [37]. Additionally, there is increased protection from ultraviolet radiation in dark-skinned individuals, who have cyclobutane pyrimidine dimer levels occurring superficially [34]. 

Lower Incidence of Sunburn Due to Higher Melanin Levels

The photoprotective effect of melanin is well-established in dermatological literature. The pigment absorbs UV radiation, preventing it from penetrating as much into the deeper skin layers where it can damage DNA. This explains the reduced incidence of sunburn in people with darker skin, who are generally less susceptible to the acute inflammatory response triggered by UV exposure [6]. It has been demonstrated that those with darker skin have significantly longer exposure times to UV radiation before sunburn results [32]. 

Additionally, the cells of darker-skinned individuals also develop fewer sunburn cells - those that die through a mechanism called apoptosis (effectively programmed cell death) due to UV-induced DNA damage. Thus, the skin of persons with darker skin is more resistant based on the direct effects of UV radiation to damage in the short term [19]. Although the risk of long-term UV-induced damage is significantly lower in dark-skinned individuals, they are not immune to other forms of photodamage.

Potential for Vitamin D Deficiency in Low UV Environments

Dark skin has the advantage of having higher amounts of melanin; this better protects against ultraviolet radiation. But it is also linked with vitamin D deficiency. It is synthesized in the skin upon exposure to ultraviolet radiation. High levels of melanin decrease the skin's capacity to generate vitamin D from sunlight. Consequently, those with darker skin are at greater risk of vitamin D deficiency, especially in regions with lower sunlight exposure, specifically regions of limited sunlight exposure in winter seasons [48]. Vitamin D deficiency is common in African ethnicity compared to white Caucasians [48]. A moderate increase in a standard erythema dose can correct the vitamin D deficiency [49].

Studies have shown that with darker skin, vitamin D deficiency becomes a significant health problem for people living in less sunny countries, particularly during winter months [50]. There is a notable long-standing association between vitamin D and various health problems like rickets and osteomalacia (impaired bone health), altered immune function, and higher cardiovascular disease risk [51]. Thus, though darker-skinned individuals benefit from protection against sunburn and skin cancer, they are at higher risk of vitamin D deficiency. To have enough vitamin D, they need a diet high in vitamin D and may require taking supplements [51].

Delayed Detection of Skin Conditions in Dark Skin Individuals

Challenges exist in diagnosing skin conditions like melanoma due to subtle or atypical presentations in darker skin tones. Dark skin individuals having higher melanin content offer increased protection against UV radiation and reduced risk of sunburn, photoaging, and UV-induced DNA damage [52]. However, elevated melanin levels do not fully protect against the development of skin conditions, such as skin cancer, and pose challenges in detecting such conditions. These challenges in detecting skin conditions stem from both the natural protective barrier nature of melanin and social and medical factors that influence the recognition of abnormalities within dark-skin individuals. Primarily, the inherent difference in pigmentation of dark-skin individuals is the reason for delayed detection of skin conditions [33]. As melanin absorbs UV radiation, it reduces the visible effects of UV exposure, such as sunburn, which are more easily detected in lighter skin. Dark-skinned individuals may not show signs of early skin damage, which makes it challenging to detect problems like sunburn, hyperpigmentation, and precancerous lesions; this is caused by elevated melanin levels causing increased pigmentation, which effectively disrupts the appearance of lesions and skin texture [33,52].

Early physical signs of melanoma include asymmetry, border irregularity, color variation, and diameter changes, which are more difficult to detect in dark skin, as pigmentation may mask these characteristics [33]. In areas where pigmentation is more uniform, melanoma lesions may not appear as distinctly different from surrounding skin, effectively complicating diagnosis [33]. This lack of visibility leads to delays in seeking medical services.

Furthermore, skin cancers like melanomas are present differently in dark-skinned individuals than in light-skinned individuals; this may lead to dark-skinned individuals being misdiagnosed or underreporting of skin conditions due to differences in how abnormalities present [53]. Melanoma in dark skin may appear as dark spots or nodules that blend into the surrounding skin. The melanoma will likely appear as an irregular and multicolored lesion in light skin. These discrepancies can cause misinterpretation of a lesion as benign or being overlooked altogether. Historically, medical research primarily focused on light-skinned individuals, leading to a lower level of awareness about risks faced by dark-skinned individuals [53]. This leads to delayed diagnosis due to a lack of regular skin checks and early intervention protocols for skin conditions.

Delayed detection of skin conditions, like skin cancer, in dark skin individuals has skewed research data. Research shows that melanoma is less common in dark skin, but it tends to be diagnosed at later, more advanced, and more difficult-to-treat stages as compared to light-skinned individuals [53]. Utilizing education campaigns to highlight the importance of regular skin checks and the need for early recognition of skin abnormalities will address the delays in the detection of skin conditions within not only dark-skinned individuals but also light-skinned individuals.

Impact of UV exposure on skin aging 

Aging of the skin is multifactorial, and it is triggered by several factors. Intrinsic aging is affected by one’s genetics and the passage of time. Additionally, extrinsic aging is determined by environmental factors, primarily UV radiation [54]. Photoaging signifies the cumulative exposure of UV radiation on the skin in a person’s lifetime, leading to physical changes over time. Variances in skin types, specifically between lighter and darker skin tones, play a significant part in how photoaging is unveiled [45]. These differences are predominantly ascribed to differences in melanin content, dermal structure, and vulnerability to UV damage.

Photoaging in Light Skin

Being exposed to UV radiation leads to an increase in oxidative stress, which leads to cellular damage. Reactive oxygen species (ROS) that are produced by UV rays cause damage to DNA, proteins, and lipids, causing disturbances in cellular function and aiding in the premature aging of skin [55]. One of the fundamental pathways of photoaging in light skin is the activation of matrix metalloproteinases, a group of enzymes that degrade collagen and elastin. Collagen and elastin are critical factors in the skin’s extracellular matrix. They provide strength, elasticity, and structural integrity to the skin [54].

Fisher et al. determined that even one dose of UV radiation can stimulate matrix metalloproteinases. This then leads to the destruction of collagen type I and III [3,56]. With time, recurrent exposure to UV rays diminishes the skin’s collagen content, blunting its capacity to repair damage. This leads to the formation of fine lines, wrinkles, and decreased skin elasticity.

Photoaging in light skin commonly manifests in the premature development of dynamic and static wrinkles. Dynamic wrinkles are formed from repeated facial expressions, meanwhile, static wrinkles progress as the skin loses its capacity to recover from creasing [3,56]. The typical areas of wrinkle formation involve the periorbital region (crow’s feet), forehead, and nasolabial folds.

In addition to wrinkles, light skin is disposed to pigmentation abnormalities caused by UV-induced damage to melanocytes. Chronic exposure to UV rays affects the function of melanocytes. This leads to irregular melanin production which is evident in solar lentigines (age spots), melasma, and additional types of hyperpigmentation [52]. Contrasting with darker skin, which is aided by the antioxidant properties of melanin, light skin is deficient in the adequate pigmentation necessary to neutralize the destructive effects of UV radiation [57]. Additionally, the vascular system of light skin is susceptible to photoaging. UV exposure can cause telangiectasia, erythema, and an uneven skin tone.

UV radiation triggers an inflammatory reaction in light skin [44]. This is evidenced by the release of pro-inflammatory cytokines such as interleukin-1 and tumor necrosis factor-alpha. These cytokines increase the effect of oxidative stress and lead to additional collagen degradation. Moreover, chronic inflammation hastens the depletion of dermal fibroblasts, which are the cells that produce collagen and elastin. Over time, this leads to the decreased resilience of the skin and intensifies the signs of aging [58].

Photoaging in Dark Skin

Dark skin, which is commonly Fitzpatrick skin types IV to VI, comprises increased levels of eumelanin, a pigment with greater UV absorbing and free radical-scavenging capabilities [1,2]. Eumelanin aids in the prevention of DNA damage that UV radiation leads to. Additionally, eumelanin decreases the activation of matrix metalloproteinases. Due to these factors, people who have darker skin commonly have delayed and signs of photoaging to a lesser extent as opposed to people who have lighter skin [55].

Taylor et al. found that eumelanin in dark skin is more effective in absorbing UV radiation [59]. This reduces the production of reactive oxygen species by up to 50%. This provides a photoprotective effect that reduces the destruction of collagen and elastin in the skin. Consequently, this leads to a delay in the production of wrinkles and the sagging of skin. Furthermore, darker skin has a tough epidermal barrier, which increases its capacity to preserve moisture and is protective against possible environmental damage [59].

In contrast with light skin, dark skin has a decreased susceptibility to the development of fine lines and wrinkles. This variance is mainly accredited to the dense collagen arrangement in the dermis of dark skin. Studies have indicated that the dermis in dark skin comprises increased levels of type I and III collagen, in addition to the increased activity of fibroblasts [60,61]. These physical advantages aid in preserving the elasticity and firmness of skin, which diminishes the manifestation of wrinkles.

Nevertheless, while dark skin is more resistant to wrinkling [61], it is more vulnerable to age-related alterations in texture and pigmentation. Over time, dark skin can develop a varicolored or irregular appearance owing to changes in melanocyte activity. Post-inflammatory hyperpigmentation is a frequent issue in dark skin [62]. It can be caused by acne, eczema, or small injuries. Post-inflammatory hyperpigmentation happens when melanocytes produce too much melanin in response to inflammation. This consequently leads to dark spots that can remain for years.

While the dermal matrix in dark skin remains reasonably well with age, there are other challenges faced such as increased pigmentation and texture [16,63]. These changes are usually intensified by UV exposure, stressing the necessity for sun protection in those with darker skin.

Although dark skin gains certain advantages from the protective effects of melanin, it is not invulnerable to the costs of UV radiation. Persistent sun exposure can nevertheless cause cumulative photodamage [24,29]. This is evidenced by fine lines, sagging skin, and irregular pigmentation.

Furthermore, dark skin is disposed to the formation of keloids and hypertrophic scars [52]. This happens when too much collagen is produced during the healing of wounds, subsequently leading to raised and thickened scars. The propensity for keloid formation in certain types of patients increases the risk of performing specific cosmetic procedures, like laser resurfacing which is used to treat photoaging. Moreover, chemical peels and microdermabrasion must be cautiously performed to reduce the risk of post-inflammatory hyperplasia and scarring in dark skin [52].

Although dark skin offers significant protection against UVB radiation, it is not entirely resistant to the effects of UVA, which penetrates deeper into the dermis. UVA exposure also contributes to the formation of reactive oxygen species, resulting in oxidative stress and collagen degradation. This process can cause subtle changes in skin elasticity and firmness.

Protective measures and recommendations 

Use of Sunscreen

The use of sunscreen has shown multiple benefits in terms of skin health in all skin types. Research has demonstrated that using sunscreen regularly not only prevents photoaging, actinic keratosis (AK), SCC, and melanoma but also prevents exacerbations of photodermatosis [64].

To minimize photoaging across all skin types, it is essential to use sunscreen with both UVA and UVB protection consistently throughout the year. The World Health Organization (WHO) advises applying sunscreen 20 minutes before sun exposure, reapplying every two hours, and reapplying again after swimming or bathing [64]. The effectiveness of a sunscreen is measured using the term “Sun Protection Factor” or SPF, which indicates the duration for which a sunscreen can shield the skin from UV radiation [47]. Research suggests that SPF testing should be conducted with an application thickness of 2 mg/cm², as applying less than this amount is linked to a reduction in the SPF. Additionally, environmental factors such as physical activity, swimming, sweating, clothing type, and contact with beach sand can influence the amount of sunscreen applied and, consequently, its effectiveness [65].

The choice of sunscreen depends upon adaptability to skin phototype, along with altitude and latitude. Ultraviolent A protection is important for all irrespective of skin type, especially in people with dark skin. To prevent photoaging caused by UV radiation, sunscreen with at least SPF 30 is generally advised for all skin types [65].

Individuals with darker skin tones are advised to use sunscreen with SPF 30+ and an SPF/UVA-Protection Factor (PF) ratio of less than 1.5, while those with lighter skin should opt for SPF 50+ with an SPF/UVA-PF ratio of less than 3 [46]. The United States Preventive Services Task Force (USPSTF) and the Food and Drug Administration (FDA) recommend using broad-spectrum sunscreen with an SPF of at least 15. However, the American Academy of Dermatology (AAD) suggests using sunscreen with an SPF of 30 or higher [66].

Furthermore, even though darker skin individuals have a decreased risk of sunburns, photocarcinogenesis, and photoaging as compared to lighter skin individuals according to research studies, a multi-modal photoprotection approach is recommended for all skin types including darker skin groups to prevent these complications [67]. 

Tailored Skincare Routines for Different Skin Types

DNA damage and accelerated aging are two negative impacts of UV radiation exposure to the skin. Numerous studies have shown a clear correlation between skin melanin levels and vulnerability to these effects. A rigorous photoprotection routine, comprising sunscreens with strong UVA-PF protection in conjunction with antioxidant formulations, is especially necessary for people with lower melanin concentrations [44].

In their investigation of the connection between skin pigmentation and vulnerability to UV-induced damage, Del Bino et al. [44] introduced the Individual Typology Angle (ITA) as a sensitivity indicator. Their research indicates that those with lighter skin and higher ITA levels need to take extra care, including using DNA-repairing products, broad-spectrum sunscreens, and intense hydration. Darker skin tones, on the other hand, have to concentrate on avoiding post-inflammatory hyperpigmentation.

The impact of high-SPF sunscreens in lowering UV-induced cellular damage was investigated by Cole et al. [47]. Lighter skin had higher amounts of cellular biomarker damage, according to their findings, highlighting the need for broad-spectrum sunscreen usage even for low levels of sun exposure. MMPs are a group of enzymes activated by UV radiation that contribute significantly to the degradation of collagen and other structural proteins in the dermis, leading to visible signs of photoaging such as wrinkles, laxity, and uneven texture. UV-induced oxidative stress upregulates MMPs, particularly MMP-1, MMP-3, and MMP-9, which break down type I and III collagen fibers in the skin [68]. This process is more pronounced in individuals with lower melanin levels, who are more susceptible to UV penetration. Photoprotection strategies, including broad-spectrum sunscreens and antioxidant-enriched skincare, have been shown to mitigate MMP activation and preserve dermal integrity. Therefore, targeting MMPs through preventive measures and topical formulations is a vital aspect of maintaining skin health across all phototypes [68].

According to skin type, D'Orazio et al. [6], described particular photoprotection measures:

Fair skin (Fitzpatrick I-II): Topical antioxidants including niacinamide and vitamins C and E, as well as daily protection with SPF 50+ and strong UVA-PF, are advised. Hyaluronic acid and ceramides are important components of hydration. Wearing hats and protective clothes can help prevent prolonged exposure to the sun.

Medium skin (Fitzpatrick III-IV): SPF 30-50 and moisturizing products like allantoin and panthenol should be used for photoprotection. To avoid hyperpigmentation, moderate exfoliation is recommended.

Dark skin (Fitzpatrick V-VI): SPF 30 sunscreens and antioxidants like vitamin C and green tea extract are necessary to prevent hyperpigmentation. Emollients' necessary fatty acids should be a part of hydration.

Young and Fajuyigbe [23] looked at how melanin modulates photobiological effects. Melanin offers some inherent defense against UV-induced DNA damage, although this defense is not complete. Thus, broad-spectrum sunscreens continue to be crucial for avoiding oxidative stress and long-term photodamage.

Gloster and Neal [33] proposed that moisturizers with extra UV filters might provide sufficient protection and support skin health. Furthermore, Gollogly et al. [69] stressed that to combat oxidative damage, customized skincare should have antioxidants such as vitamins C and E. Collagen-enhancing ingredients such as peptides and retinoids may help fair-skinned people seem less wrinkled and fine-lined after sun exposure. On the other hand, hyaluronic acid and niacinamide may be useful for those with darker skin tones in preventing hyperpigmentation and maintaining enough moisture.

High-SPF, broad-spectrum sunscreens should be the top priority in tailored skincare, especially for people with fair skin. To strengthen the defense against oxidative damage, those with dark or olive complexions can use antioxidants like vitamins C and E [70].

For lighter skin types, which are more vulnerable to sunburn and DNA damage, Passeron et al. [46] emphasized the need to apply high-SPF sunscreens with excellent UVB protection. Sunscreens with iron oxides and antioxidants are advised for darker skin types since they offer defense against UVA rays and visible light, which can aggravate pigmentation disorders like melasma.

## Conclusions

By understanding the fundamental differences in the structural integrity of skin types, we better evaluate the levels and types of photodamage that occur across the spectrum. This, in turn, allows for the individualized tailoring of skin regimens to maximize effective results. It is established that one critical difference between light and dark skin is the comparative levels and distribution of pheomelanin and eumelanin respectively; the latter of which is more quickly photosensitized to produce higher amounts of ROS. This means that darker skin allows less than half of UVA and UVB absorption compared to lighter skin. Hence, increased prevalence of skin cancers is observed in the lighter-skinned population, especially those with chronic or high episodic exposure in geographical areas distant from the ancestral homelands.

Increased oxidative stress leads to cellular damage causing premature aging of the skin. Activation of matrix metalloproteinases degrade collagen and elastin which over time can dimmish the skin’s capacity to repair damage, causing fine wrinkles and decreased elasticity. In darker skin this leads to irregular melanin production presenting as solar lentigines, melasma and types of hyperpigmentation. In light skin the exposure can cause telangiectasia and erythema. Tailored skin regimens include daily protection with SPF 50+ in lighter skin and SPF 30-50 in darker skin. While avoiding hyperpigmentation is the main focus of management in darker skin types, topical antioxidants, hydration and prevention are important to all skin types in minimizing damage. 
